# Fabrication and Characterization of Wrapped Metal Yarns-based Fabric Temperature Sensors

**DOI:** 10.3390/polym11101549

**Published:** 2019-09-23

**Authors:** Qian Yang, Xi Wang, Xin Ding, Qiao Li

**Affiliations:** 1 Key Laboratory of Textile Science & Technology of Ministry of Education, College of Textiles, Donghua University, Shanghai 201620, China; 2 Engineering Research Center of Digitized Textile & Apparel Technology, Ministry of Education, College of Information Science and Technology, Donghua University, Shanghai 201620, China

**Keywords:** temperature sensor, metal fiber, wrapping technology, mechanical performance, response time

## Abstract

Textile temperature sensors are highly in demanded keep a real-time and accurate track of human body temperature for identification of healthy conditions or clinical diagnosis. Among various materials for textile temperature sensors, temperature-sensitive metal fibers have highest precision. However, those metal fibers are mechanically too weak, and break constantly during the weaving process. To enhance the mechanical strength of the metal fibers, this paper proposes to make wrapped metal fibers using wrapping technology, and characterize the effect of wrapped metal yarns on both mechanical properties and sensing behaviors. The wrapped yarns were woven into fabrics, forming the fabric temperature sensors. Results show that strength and maximum strain of the wrapped yarns are 2.69 and 1.82 times of pure Pt fibers. The response time of fabric temperature sensors using wrapped yarns was observed as 0.78 s and 1.1 s longer compared to that using Pt fibers when front and back sides contacted heat source, respectively. It is recommended that the wrapping method should be implemented for the protection of Pt fibers in fabric temperature sensors.

## 1. Introduction

Textiles are ideal hosts for electronic devices when electronics are applied to the human bodies due to their favorable flexibility and comfort [1]. Currently, there is growing interest in intimately wearable electronics or next-to-skin health monitoring networks or systems [2,3]. Fabric-based electrodes and sensors are consistently used to provide physiological information in various health-care applications [4,5] or real-time performance feedback to athletes and coaches in competitive sport [6,7].

As a basic physiological signal, body surface temperature reflects the temporal state of heat transfer between the human body and the environment [8]. Accurate and real-time detected skin temperature in human activities has been reported as being of great importance when analyzing and identifying sophisticated health conditions for clinical diagnosis [9,10], such as cardiovascular diseases, pulmonological diagnostics, and other syndromes [11,12]. There are numerous medical temperature sensors. Besides conventional mercury thermometers, thermocouple thermometers, and infrared thermometers, novel temperature sensors, such as optical temperature sensors, have been proposed and are attracting substantial attention due to their favorable functions [13,14,15]. However, such temperature sensors are generally rigid and bulky in size, hence always limited in in-lab use, instead of being introduced in wearable applications, where a conformal or flexible interface is much demanded between the sensors and the soft and curved human skin with minimal user awareness or discomfort [16,17,18].

To satisfy wearable measurements of human skin temperature, much effort has been devoted to developing various types of flexible temperature sensors, which can be primarily categorized into three groups, i.e., polymeric sensible sensors, metal films on compliant substrates [19,20,21], and temperature-sensitive metal fibers in textile structures. The polymeric sensors, formed by filling conductive particles into a polymer matrix, detect temporal temperature based on the difference in thermal expansion coefficients between the conductive fillers and the soft polymers [22,23,24]. Polymeric temperature sensors have high sensitivities. It has been demonstrated that electrical resistance of the conductive composites changes exponentially with temperature [25]. However, it is still challenging to fabricate a polymeric sensor with a reproducible and accurate sensing response to temperature, especially for a required working range of temperature for wearable applications [26]. The other two alternative temperature-sensitive materials or structures integrate temperature-sensing materials or elements, such as metal films or free-standing conductors, into compliant substrates such as polydimethylsiloxane (PDMS), polyimide, papers, as well as textiles [27,28]. The major merits of these kinds of temperature sensors are high precision, good reproducibility and fast response rate in the real-time detection of skin temperatures [29]. The authors’ group has also proposed the design of a textile-based temperature sensor by integrating a continuous metal fiber into a woven-structured fabric based on conventional weaving technology [30]. The manufactured samples of fabric temperature sensor operate based on the almost linear correlation between resistance of the metal fiber and the localized temperature, which can be expressed by R = R_0_ × (1 + α(T − T_0_)), where R and R_0_ are resistances of the metal fiber at temperature T and reference temperature T_0_, respectively; α is temperature coefficient of resistance (TCR), which is a constant with value of 0.0039 °C^−1^. This fabric temperature sensor has demonstrated high accuracy (error: ±0.2 °C), superior resolution (0.05 °C), fast response, and almost no hysteresis. However, metal fibers with ultra-fine diameters are mechanically weak and break constantly, making the textile manufacturing processes rather difficult [31,32].

Previous studies have applied metal fibers to actual textiles by spinning metal fibers and ordinary textile materials together [33,34,35,36,37,38], producing a cotton/metal composite yarn in a modified ring spinning machine [39,40]. A complex yarn contains metal fibers by using a ring-spinning machine. It has been demonstrated that the tenacity and hairiness of the complex yarns is affected by various parameters (such as core materials, roving materials, twist level and count of the spun yarn) [41]. Hence, to protect the metal fibers from mechanical and electrical failure in the weaving process, we propose wrapping textile yarns around the metal fibers and would specifically consider the changes in mechanical properties of the metal fibers, since the above works mainly focused on the whole complex yarn.

Hence, this article provides a new perspective of mechanical enhancement of the metal fibers through wrapping technology, and then involves wrapped metal yarns in the production of the fabric temperature sensors via weaving technology. First, the wrapped yarns consisting of metal fiber as a core yarn and two covered layers were manufactured and tested. Then, the wrapped yarns were woven into fabrics, forming the fabric temperature sensors. Results show that the response time of the temperature-sensing element after wrapping is relatively acceptable. Therefore, it is recommended that wrapping method shall be implemented for the protection of the metal fibers in fabric temperature sensors.

## 2. Experiments

### 2.1. Fabrication of Wrapped Yarns

**Figure 1:**
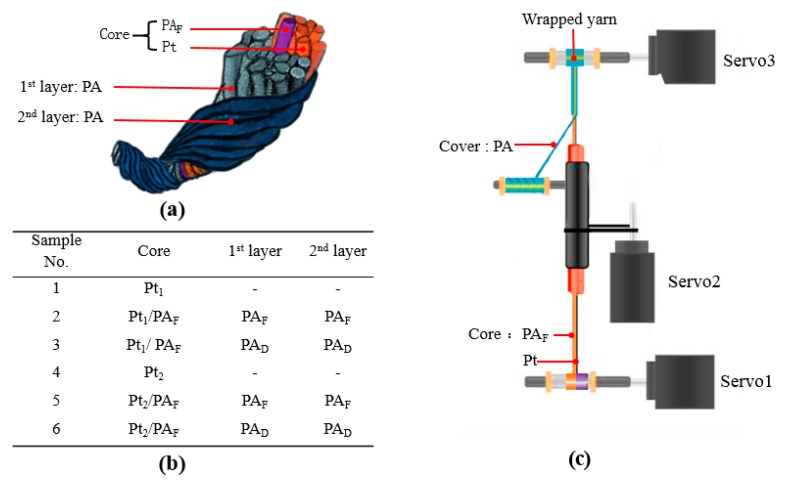
Pt/PA 66 wrapped yarns. (**a**,**b**) Structure and types of the wrapped yarns; (**c**) Fabrication process. Note: Pt_1_ and Pt_2_ are the Pt fibers with diameters of 20 μm and 30 μm, respectively. PA_F_ means PA-FDY; PA_D_ means PA-DTY.

To improve the mechanical performance of the pure metal fibers, yarn-covering technology was employed by wrapping multi-filaments around the core metal fibers. As illustrated in Figure 1, different types of wrapped yarns consisting of core metal fibers and two covering layers of multi-filaments were produced by a self-modified hollow-spindle spinning machine. As shown in Figure 1a,b, we used platinum (Pt) fiber as the temperature-sensitive material owing to its desirable thermal properties, antioxidation, linearity between electrical resistance, and temperature. Two sizes of Pt fibers with diameters of 20 μm and 30 μm, obtained from Shanghai Shengjie Instrumentation Ltd., Shanghai, China, were selected and used as the core fiber. Polyamide 66 (PA) multi-filaments with linear density of 70 Denier (D), provided by Shanghai Qingxi Chemical Fiber Technology Co., Ltd., Shanghai, China, were selected as the wrapping elements either in the form of fully drawn filament yarn (FDY) or drawn textured yarn (DTY). Different types of covered yarns were fabricated by wrapping the PA66 filaments around the core fiber via a self-modified hollow-spindle spinning machine. As plotted in Figure 1c, feed roller, hollow spindle, and the winding roller were controlled by three servos. Servo1 and servo3 have the same speed, and the winding speed can be determined by the ratio of the rotation speed of servo1 to servo2. Due to the poor elongation of the Pt fiber, the core part also involves PA 66multi-filaments, except for Pt fiber. PA 66 filaments in the form of Z twist constitute the first covering layer, while PA 66 filaments with S twist make up the second wrapping layer.

### 2.2. Characterization of Wrapped Yarns

Morphological observation of the samples was conducted with the TM-3000 scanning electron microscope (Hitachi, Japan). 

**Figure 2:**
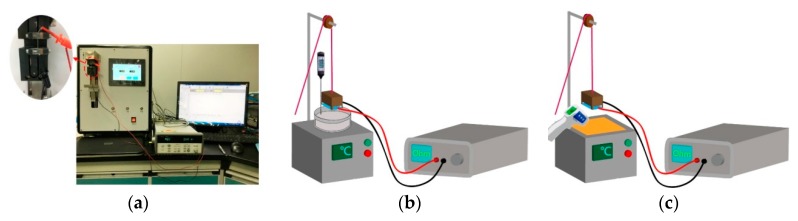
Experimental setup for tensile test of the wrapped yarns (**a**); and resistance change with different temperature in oil-bath (**b**) and hotplate stage (**c**).

Tensile tests were conducted on all the four types of samples—five specimens for each type. The experimental setup is shown in Figure 2a. The specimen was fixed on two clamps of the XQ-2 fiber strength meter (provided by Shanghai New Fiber Instrument Co., Ltd., Shanghai, China) with a gauge length of 20 mm and a pre-tension of 0.1 N. The crosshead is 200 cN, while extension rate was set to 10 mm/min. During the stretch test, the electrical resistance of the metal fibers was monitored by Agilent 34970A. Strength and maximum strain of the wrapped yarns were specifically examined.

The electrical resistance of all the four types of samples with temperature variation was investigated in an oil-bath and on a hotplate. The temperature was adjusted in a range from 30 °C to 50 °C. The resistance of the samples was recorded by Agilent 34970A, provided by Agilent Technologies Inc., Santa Clara, USA (Figure 2b,c).

### 2.3. Fabrication and Characterization of Fabric Temperature Sensors

**Figure 3:**
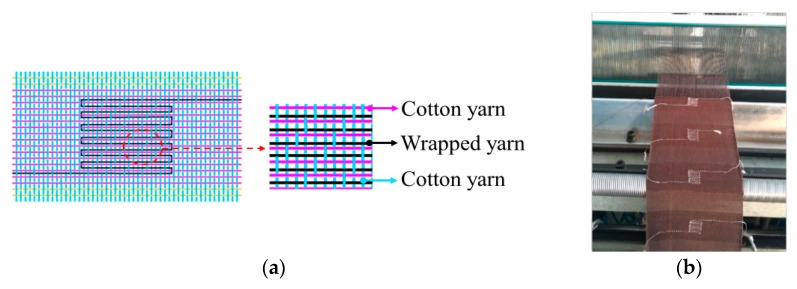
Fabrication of fabric temperature sensors. (**a**) Structure of the fabric sensors; (**b**) Fabrication process.

An effective design of temperature-sensing fabric is to arrange the samples of the wrapped yarns in serpentine patterns, in consideration of the initial resistance and requirements for external circuits. Hence, a continuous wrapped yarn was woven in an organized floating pattern into a fabric composed of cotton yarns as both transverse and longitudinal elements using an automatic weaving machine (Figure 3). The one-third right twill was chosen as basic structure, with weft density of 200 threads per 10 cm and warp density of 210 threads per 10 cm. The sensing area is 100 mm^2^ in the center of the fabric, led by two wires at a length of about 25 mm for electrical connection to the outer circuits or components.

Morphological images of the fabric temperature sensors were observed under HDMI200C-B electron microscope (Shenzhen ZongyuanWeiye Technology Co. Ltd., Shenzhen, China). 

The electrical resistance of all the fabric sensors with temperature variation was investigated in an oil-bath and on a hotplate, as mentioned above.

## 3. Results and Discussion

### 3.1. Pt/PA Wrapped Yarns

**Figure 4:**
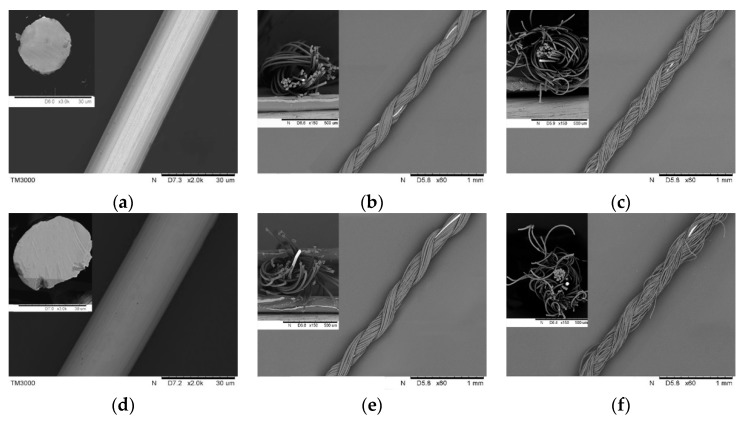
SEM images of the metal fibers and their wrapped yarns. (**a**–**f**) Samples from No.1 to No.6, respectively.

Four types of covered yarns with 1000 twist/m were finally obtained. Figure 4 shows the scanning electron microscope (SEM) images of all the samples from pure metal fibers to the different wrapped yarns. The free-standing Pt metal fibers (Young’s modulus: 160GPa, Poisson’s ratio: 0.38), with two different diameters of 20 μm (Figure 4a) and 30 μm (Figure 4d), respectively, have a smooth surface and circular-like cross-sections. The electrical resistance is 3.79 Ω·cm^−1^ and 1.70 Ω·cm^−1^; the tensile strength is 20.26 cN and 27.40 cN; the maximum strain is 1.04% and 0.90% for both two Pt fibers. Both were covered by different PA filaments, i.e., PA_F_ and textured PA (PA_D_) yarns. From their SEM pictures, the Pt metal core is almost completely covered by two layers of PA filaments and the yarn twist is approximately uniform. The samples with PA_D_ filaments have an average diameter of 220 μm, which is a little larger than those with PA_F_ filaments, whose mean diameter is 190 μm. This can also be confirmed from their cross-sectional views, where the wrapped yarns with PA_D_ filaments have a looser structure while the core is more tightly packed in those with PA_F_ layers. In addition, observed from both axial and cross-sectional views, the core metal fiber has a spiral path in the whole structure, and a small segment is exposed on the surface of the yarn, in particular, with the PA_F_ filaments as the outer layer. This phenomenon is intentionally produced by adjusting the yarn tension in the fabrication process for the increment of the tensile strain of the metal fiber.

**Figure 5:**
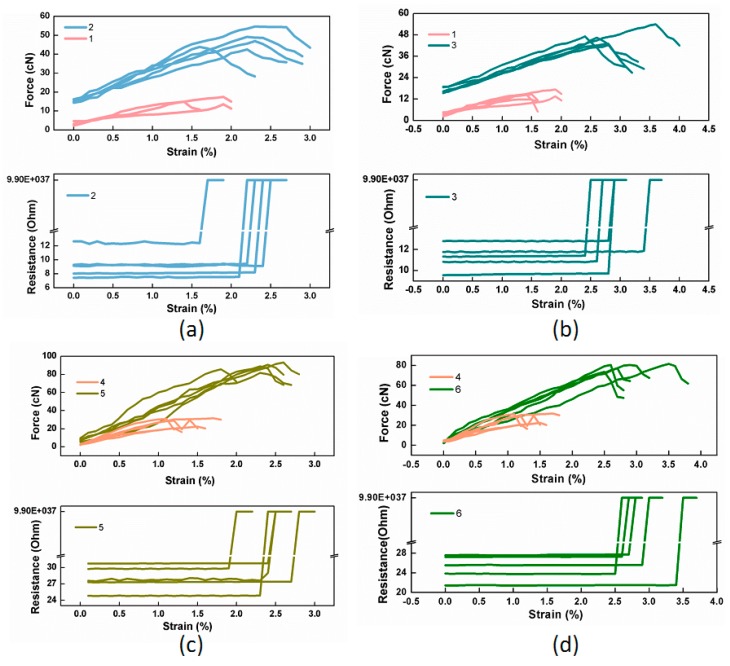
Tensile performance of all the samples. (**a**–**d**) Force-strain-resistance curves of all the samples from No.1 to No.6 at electrical failure.

To examine whether the mechanical performance of the pure Pt fibers has been enhanced by being covered with PA filaments, samples of wrapped yarns underwent a tensile test as elaborated above. The observed load-strain-resistance relations of all the samples are plotted in Figure 5, from which the critical force and strain at electrical failure are identified and summarized in Table 1. The breaking force of the pure metal fiber with 20 μm diameter is 17.79 (±0.56) cN, dramatically increasing to 47.36 (±2.39) cN and 46.58 (±2.33) cN at electrical failure after being wrapped by PA_F_ and PA_D_ filaments, respectively. An increase of critical strain is also observed as raised from 1.16% to 2.12% and 2.84%. Similarly, the metal fiber with a diameter of 30 μm has a significant increment in its critical load from 31.31 (±0.75) cN to 88.06 (±2.24) cN and 78.57 (±1.69) cN and corresponding elongation from 1.13% to 2.28% and 2.84% at electrical failure via yarn-covering method by both PA_F_ and PA_D_ filaments. Hence, it can be concluded that the yarn-covering technology has had a positive effect on the mechanical performance of the pure metal fibers due to their spiral shape in the wrapped structure. During the whole process, the curved pure metal fiber was straightened first before it could undertake tension. Additionally, when PA_F_ filaments were used as covered layers, the critical load was 2.69 and 2.81 times of those of Pt fibers with different diameters; their corresponding elongation was 1.82 and 2.02 times of pure Pt fibers. By contrast, when PA_D_ filaments are taken as the outer layers, the load was 2.62 and 2.53 times; their strain was 2.45 and 2.52 times of both pure metal fibers. The slight discrepancy in mechanical enhancement of the metal fibers may be attributed to the tight and loose structures of the PA filaments in the wrapped yarns, where the later could bear a little larger strain with a smaller force due to their textured filaments.

**Figure 6:**
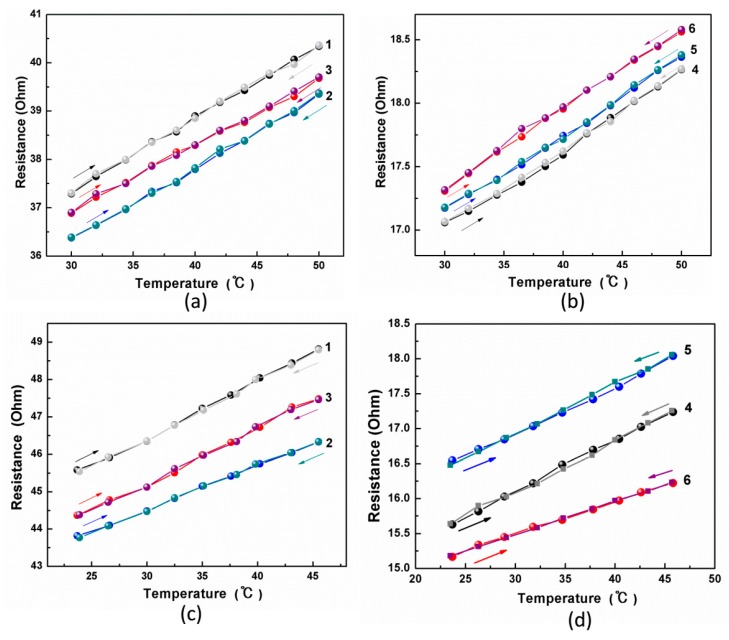
Temperature-sensitive performance of all the samples. (**a**–**d**) Resistance-temperature curves of all the samples from No.1 to No.6 in the conditions of oil bath (**a**,**b**) and hotplate stages (**c**,**d**).

To see whether the temperature-sensitive performance of the pure metal fiber is influenced by the wrapped structure, the relative resistance variation with temperature was investigated. Both a temperature-controlled oil bath and digital hotplate were used to control the temperature ranging from 30 °C to 50 °C, which is consistent with human body temperature range. The resistance-temperature curves of all the samples are plotted in Figure 6. It can be observed that for each sample, the electrical resistance raised almost linearly while temperatures increased from 30 °C to 50 °C with either oil bath or hotplate. The coefficients of linearity r^2^ were calculated through linear fitting and summarized in Table 2. All the obtained coefficients of linearity surpassed 0.999, suggesting that the yarn-covering technology has no negative effect on the linearity between the resistance and temperature of the pure Pt fibers. The TCRα, i.e., the slope of resistance-temperature curves of all the samples was calculated and is summarized in Table 2. All the TCR values generally maintained (with an averaged value of 0.00358 °C^−1^), suggesting there is no significant difference in sensitivity between the wrapped yarns and the pure Pt fibers. Those experimental data demonstrate that the temperature-resistivity characteristics of the wrapped yarns remain at the same level as those of the free-standing metal fibers since the mechanism of the temperature sensor is only based on the changes of the metal resistance when subject to the temperature variation. In addition, the slopes of the resistance-temperature curve for both heating and cooling processes were exactly the same, suggesting that there was nearly no hysteresis during the loading-unloading cycle of temperature. 

The response time, i.e., the time required for the electrical resistance of the sensor to achieve 63.2% of its final value when subjected to a step change in surrounding temperature, was used to characterize the sensor response [42]. In this work, the step change of temperature was calculated by moving the samples from air at ∼20 °C into an oil bath or onto a hotplate at around 50 °C. The transient trend of resistance was observed as gradually rising exponentially with temperature, which was continuously collected by Agilent 34970A with a sampling rate of 50 ms^−1^.

**Figure 7:**
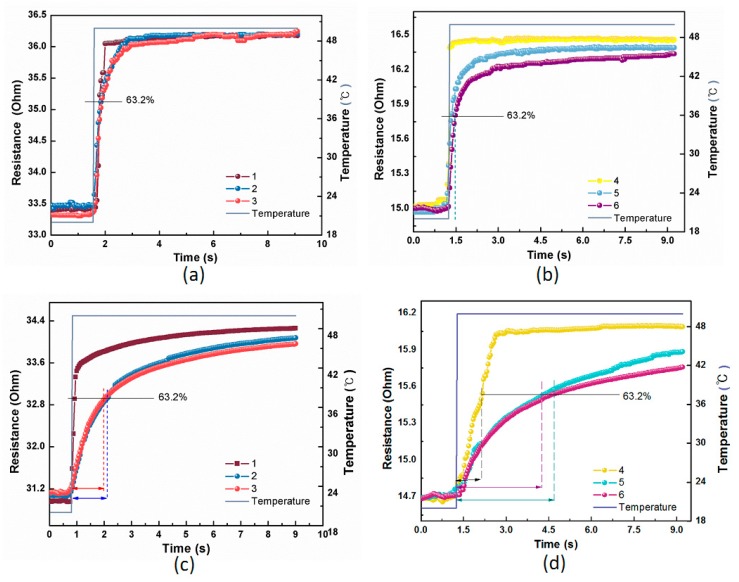
Response time of all the samples in oil bath (**a**,**b**) and on hotplate (**c**,**d**).

The response time was identified from the curve of real-time resistance response when the samples were subjected to a step change of temperature. As illustrated in Figure 7, the electrical resistance of all the samples gradually rose and reached a plateau during this sudden change in temperature. It can be observed from Table 3 that the typical response time of the free-standing Pt metal fibers is much less than 1 s either in oil-bath or hotplate conditions, while the response time of wrapped yarns became longer. This is because the PA filaments act like a thermal shield, preventing heat transfer to the pure metal fibers, yielding a slower response time with wrapped yarns. 

**Figure 8:**
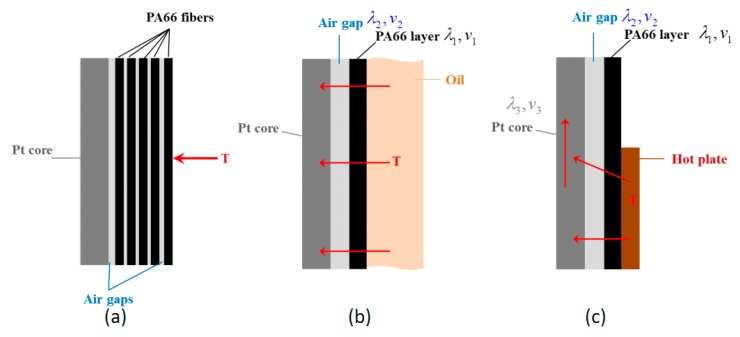
Ideal serial heat-transfer models. (**a**) Schematic heat transfer from outside to Pt core, accrossing fibers and air gaps; (**b**) Simplified model subjected to hot oil; (**c**) Simplified model heated by hotplate.

To understand the above results, an ideal serial model of heat transfer (Figure 8) was considered, where heat flow is vertical to the interface between the two components, i.e., along the direction of thickness. 

As shown in above figure, the heat transfers through the PA66 fibers and air gaps to reach the Pt core (Figure 8a) can be simplified as heat flow across the PA66 layer and equivalent air gap, when subjected to an oil bath and hotplate. Thermal conductivity coefficients and volumes of the corresponding material are denoted as λ and *v*, as shown in Figure 8. According to the equation of overall thermal conductivity coefficient of two pure materials in serial

$$\lambda =\frac{1+\frac{V_{2}}{V_{1}}}{\frac{1}{\lambda_{1}}+\frac{V_{2}}{V_{1}\lambda_{2}}}$$

it easy to see that the function of overall thermal conductivity coefficient λ shown above is an increasing function of λ_i_ as well as decreasing function of *v*_i_ when λ_i_ < 1. Hence, it can be concluded that the resultant thermal conductivity for wrapped yarns (PA66+Pt) is always lower than pure Pt fibers, which explains a longer response time of wrapped yarns. Moreover, as shown in Figure 8b,c, the involved components for the wrapped yarns are Pt, PA66 layer, and air. As can be seen from Table 4, the thermal conductivity coefficient of PA66 is much larger than that of air, and both far less than Pt core [43]. In this case, the volume ratio of the two(*v*_2_/*v*_1_) in the wrapped structure is the determinant factor of the overall λ. Since the PA_D_ structure has a bigger bulk volume due to the fluffy curl shape, it keeps more air afterwards and leads to a smaller λ than that of the PA_F_ wrapped structure. Table 3 also shows that the response time of the PA_F_ wrapped structure is slightly shorter than that of the PA_D_ wrapped structure. Moreover, Figure 8c also explains that for wrapped yarns subjected to a hotplate, longer response time was observed mainly due to the partial contact between hotplate and PA66 layer.

**Figure 9:**
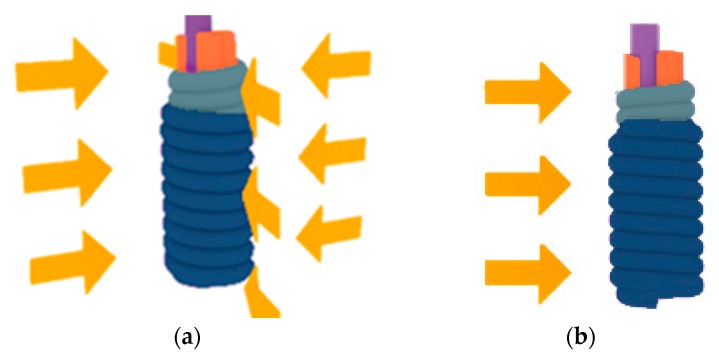
Heat-transfer process of the wrapped yarns in oil-bath (**a**) and hotplate conditions (**b**).

Moreover, the response rate of the wrapped yarns is much slower in the hotplate condition than that in oil bath, suggesting that the operation conditions contribute a lot to the response time of the sensor. This can be explained by Figure 9, where it can be seen that in the flowing liquid of heat convection, the yarn surface could make contact with the liquid media, which helps heat transfer easily to the core fiber, yielding a faster response time. While on a hotplate, however, due to the non-planar structure of the wrapped yarn, the wrapped yarn cannot sufficiently contact the flat mounting area, leading to a much slower response. Hence, the response time of the wrapped yarns depends on the working condition under which the sensing element is operating. Exact conditions of the test must first be specified together with the response time constant before applying the wrapped yarns into applications. 

### 3.2. Fabric Temperature Sensors

**Figure 10:**
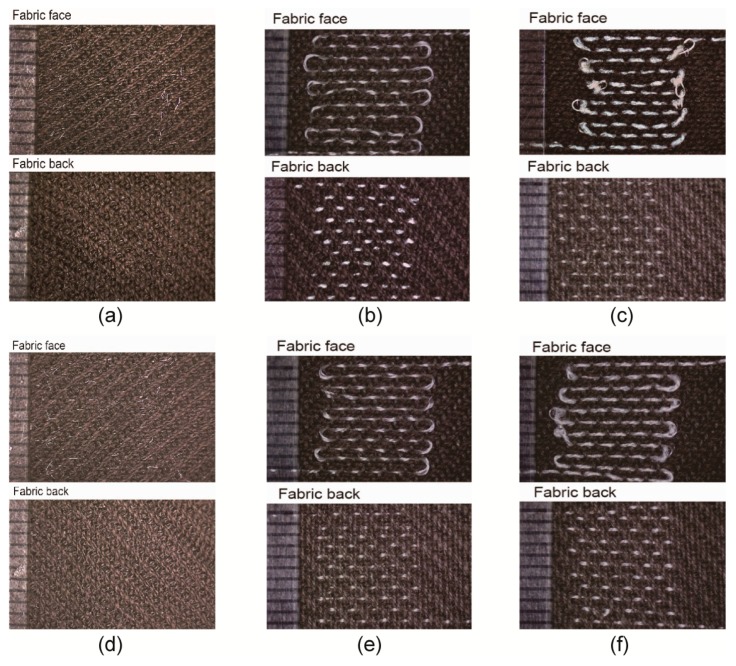
Microscopic images(X12) of all the samples of the fabric temperature sensors from No.#1(**a**) to No.#6 (**f**) with front and back sides.

Easy to see that the wrapped yarns have higher breaking tensile strength (> 40 cN), the same sensitivity, and acceptable response time compared with pure metal fibers, and thus are more suited and easier to incorporate into the woven structure for fabric temperature sensors. Hence, a wrapped yarn was woven into an organized floating pattern into a fabric composed of cotton yarns as both transverse and longitudinal elements using an automatic weaving machine. Figure 10 shows microscopic images of the samples of the fabric sensors. It can be seen that all the electrodes are almost hidden in the woven fabric with free-standing metal fibers (Figure 10a,d). A small segment of metal fibers may be exposed when using the wrapped yarn in the fabric, particularly when PA_D_ filaments were used as covering yarns. Due to the difference in bulk shape between covering yarn and core yarn, a reverse twist in the wrapped yarn would lead to a large discrepancy of PA_D_ wrapped structure, in which the core yarn is more likely to be uncovered. Since the diameter of the wrapped yarns is much larger than the pure metal fibers, the courses of the metal fiber in the sensitive area are 13 with a spacing of 800 μm and a total length of 170 mm, while the course numbers of the wrapped yarns are 11 with a spacing of 880 μm and a total length of 145 mm either covered by PA_F_ filaments or PA_D_ filaments. 

**Figure 11:**
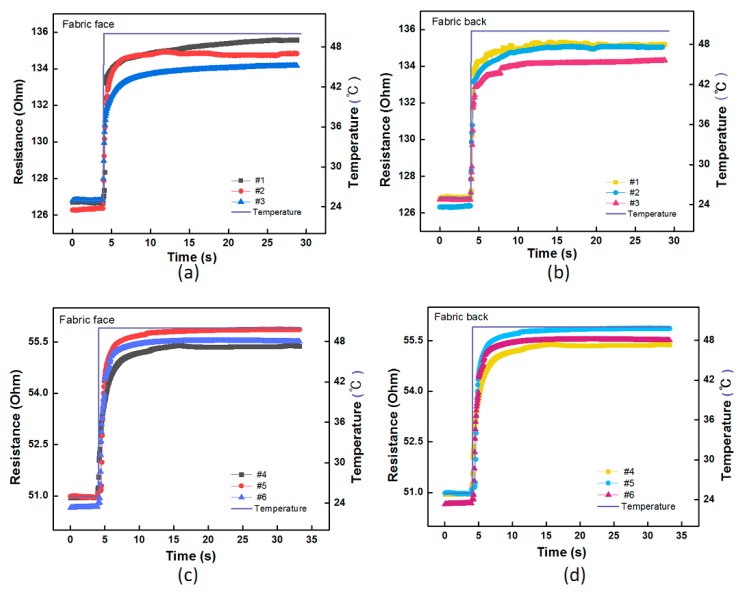
Response time of all the fabric temperature sensors in oil bath. (**a**,**b**) Resistance/temperature-time curves of the sensors with front and back sides from No.1 to 3; (**c**,**d**). Resistance/temperature-time curves of the sensors with front and back sides from No.4 to 6.

As above, the response time of the fabric temperature sensors was also characterized by moving the samples with both sides from air at ∼20 °C into an oil bath around 50 °C. The resistance of the samples was continuously measured by Agilent 34970A at a sampling rate of 50 ms^−1^. As illustrated in Figure 11, the electrical resistances of all the samples gradually rose and reached a plateau during this sudden change of temperature from 20 °C to 50 °C. The typical response time of the fabric sensors with the free-standing Pt metal fibers as well as with wrapped yarns were summarized in Table 5. It can be observed that for fabric sensors with free-standing Pt fibers, the response time was about 0.7 s in the oil-bath condition, slightly smaller than that of fabric sensors with wrapped yarns, suggesting that the wrapping method is acceptable as it does not significantly change the sensing property of the fabric sensors. This result can be explained by our previous analysis for the wrapped yarns. The silicone oil filled the holes and openings of the fabric, leading to a direct heat transfer between the silicone oil and temperature-sensitive material in the fabric. Hence, for fabrics woven with pure Pt filament, the heat-transfer process was more effective compared to that with wrapped structure, in which heat had to transfer through PA filaments, of which the thermal coefficient is far lower than the Pt fiber. Moreover, there is no obvious difference in the response time for front and back sides of the fabric sensors because the flowing liquid media could permeate and reach the Pt core in wrapped yarns from both sides. 

**Figure 12:**
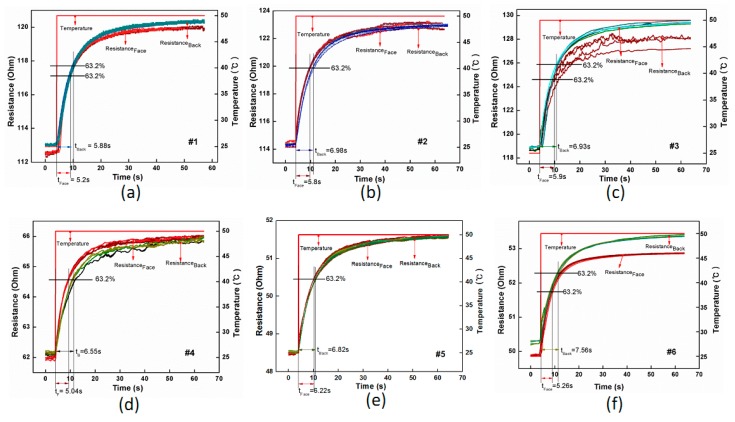
Response time of all the fabric temperature sensors on hotplates. (**a**) Sample No.1 with front and back sides; (**b**) Sample No.2; (**c**) Sample No.3; (**d**) Sample No.4; (**e**) Sample No.5; (**f**) Sample No.6.

Since the response time of the fabric temperature sensors is also affected by environment and operation condition of the sensors, samples of fabric temperature sensors underwent a sudden change of temperature by moving the samples from air at ∼20 °C onto a hotplate at around 50 °C. The resistance of the samples is continuously collected by Agilent 34970A at a scanning speed of 50 ms^−1^. As illustrated in Figure 12, the electrical resistance of all the samples gradually rose and reached a plateau during this sudden change of temperature. As summarized in Table 6, the typical response time of all the fabric temperature sensors is much larger than that of those in oil-bath conditions. This is because in the convection liquid, the sensing material, i.e., Pt fiber, could sufficiently contact the liquid media to receive easy heat transfer, and thus a faster response time. On the hotplate, however, due to the non-planar nature of the fabric structure, the curved fabric sensor cannot fully conform to a flat mounting area, leading to a much slower heat transfer and longer response time. The response time of samples with wrapped yarns was generally larger than that with pure Pt fibers, due to the protection effect of the covering yarns. In addition, the response time is much larger when the fabric back contacts the hotplate than with front sides, due to the smaller segment of exposed contact areas of Pt/PA wrapped yarns. As a result, heating transfer efficiency from plate to fabric face is higher than that to fabric back. This explains that when the fabric back made contact with the plate, it took more time to respond than the fabric front. For instance, sample No.6 of the fabric temperature sensor with back side gives a value of 7.56 s as a maximum response time, while when its face contacts the plate it takes only 5.26 s, leading to the largest difference of front and back response time, i.e., 2.3 s.

## 4. Conclusions

In this work, metal fiber Pt with two different diameters were wrapped with PA_F_ and PA_D_ filaments to form four kinds of wrapped temperature-sensing elements, aiming to improve mechanical properties such strength and strain of the pure metal fibers. Results show that wrapping technology can effectively increase the overall bearing capacity and elongation but increased the temperature response time of the component to some extent. When the diameter of core yarn Pt is 20 μm and the cover yarn is PA_F_ filaments, the integral strength of the wrapped structure is the largest, and caused a smallest increase in response time. Compared to the pure Pt fiber with diameter of 20 μm, the bearing capacity and elongation increased by 2.69 and 1.82 times, respectively. It was also observed that in an oil bath, there was no significant difference between response times of the fabric sensor with pure Pt fiber and that with wrapped yarns. Fabric temperature sensors designed for real-time detection of skin temperature, with or without wrapping the temperature-sensitive Pt fibers, were further designed, fabricated, and tested. When using the hotplate, the response time of fabric temperature sensors with wrapped yarns was 0.78s longer and 1.1s longer than with pure Pt fibers when front and back sides were in contact with the hotplate, respectively. Given that the breaking strength and elongation when using wrapped yarns are significantly improved, and that the response time of the temperature-sensing element after wrapping is relatively acceptable, it is recommended that the wrapping method should be implemented for the protection of yarns in fabric temperature sensors.

## Figures and Tables

**Table 1:** Force and strain of all the samples at electrical failure.

Sample No.	Force (cN)	Deviation (±cN)	Strain (%)	Deviation (±%)
1	17.79	0.56	1.16	0.13
2	47.36	2.39	2.12	0.15
3	46.58	2.33	2.84	0.23
4	31.31	0.75	1.13	0.14
5	88.06	2.24	2.28	0.13
6	78.57	1.69	2.84	0.19

**Table 2:** TCR and correlation coefficients of all the samples.

Indicator	Environment	Process	1	2	3	4	5	6
α (Ω/°C)	Oilbath	Heating	0.00381	0.00378	0.00352	0.00328	0.00323	0.00337
		Cooling	0.00409	0.00410	0.00380	0.00352	0.00315	0.00365
	Hotplate	Heating	0.00409	0.00315	0.00478	0.00325	0.00322	0.00263
		Cooling	0.00394	0.00292	0.00422	0.00308	0.00301	0.00255
r^2^	Oilbath	Heating	0.99941	0.99913	0.99861	0.99783	0.99614	0.99882
		Cooling	0.99904	0.99900	0.99907	0.99876	0.99826	0.99875
	Hotplate	Heating	0.99891	0.99833	0.99928	0.99883	0.99487	0.99951
		Cooling	0.99961	0.99979	0.99836	0.99908	0.99842	0.99927

**Table 3:** Response time of all the samples in oil-bath and hotplate conditions.

Sample No.	Temperature Range (°C)	RT_O_^1^ (s)	RT_p_^1^ (s)
1	20 ± 2–50 ± 2	0.25 ± 0.03	0.28 ± 0.12
2		0.55 ± 0.05	1.56 ± 0.10
3		0.60 ± 0.04	2.28 ± 0.14
4		0.15 ± 0.05	0.96 ± 0.08
5		0.25 ± 0.10	3.92 ± 0.06
6		0.25 ± 0.05	3.68 ± 0.06

^1^ RT_o_ and RT_p_ mean response time tested in the conditions of oil bath and hotplate, respectively.

**Table 4:** Thermal conductivity coefficient of related materials.

Material	Air	PA66	Pt
λ(W/(m·°C)	0.027	0.28	72

**Table 5:** Response time of the fabric temperature sensors in oil bath with two sides.

Fabric Specimens	Temperature Range (°C)	RT_f_^1^ (s)	RT_b_^1^ (s)
#1	20 ± 2–50 ± 2	0.75 ± 0.05	0.70 ± 0.05
#2		0.75 ± 0.10	0.85 ± 0.15
#3		0.95 ± 0.10	0.90± 0.10
#4		0.70 ± 0.05	0.75 ± 0.15
#5		0.95 ± 0.05	1.08 ± 0.10
#6		1.15 ± 0.10	0.85 ± 0.25

^1^ RT_f_ and RT_b_ mean response time tested in the conditions of face side and back side, respectively.

**Table 6:** Response time of fabric sensors with face and back sides on heating plate.

Fabric Specimens	Temperature Range (°C)	RT_f_ (s)	RT_b_ (s)
#1	20 ± 2–50 ± 2	5.02 ± 0.19	5.88 ± 0.50
#2		5.80 ± 0.33	6.98 ± 0.31
#3		5.90 ± 0.30	6.93 ± 0.28
#4		5.04 ± 0.12	6.55 ± 0.47
#5		6.22 ± 0.13	6.82 ± 0.20
#6		5.26 ± 0.30	7.56 ± 0.41
